# A Sparse Probabilistic Code Underlies the Limits of Behavioral Discrimination

**DOI:** 10.1093/cercor/bhz147

**Published:** 2019-08-12

**Authors:** Balaji Sriram, Lillian Li, Alberto Cruz-Martín, Anirvan Ghosh

**Affiliations:** 1 Division of Biology, University of California San Diego, La Jolla, CA 92093, USA; 2 Research and Early Development, Biogen, Cambridge, MA 02142, USA; 3 Department of Biology; 4 Neurophotonics Center; 5 Department of Pharmacology and Experimental Therapeutics, Boston University, Boston, MA 02215, USA

**Keywords:** electrophysiology, mouse behavior, orientation discrimination, population coding, 2AFC

## Abstract

The cortical code that underlies perception must enable subjects to perceive the world at time scales relevant for behavior. We find that mice can integrate visual stimuli very quickly (<100 ms) to reach plateau performance in an orientation discrimination task. To define features of cortical activity that underlie performance at these time scales, we measured single-unit responses in the mouse visual cortex at time scales relevant to this task. In contrast to high-contrast stimuli of longer duration, which elicit reliable activity in individual neurons, stimuli at the threshold of perception elicit extremely sparse and unreliable responses in the primary visual cortex such that the activity of individual neurons does not reliably report orientation. Integrating information across neurons, however, quickly improves performance. Using a linear decoding model, we estimate that integrating information over 50–100 neurons is sufficient to account for behavioral performance. Thus, at the limits of visual perception, the visual system integrates information encoded in the probabilistic firing of unreliable single units to generate reliable behavior.

## Introduction

Animals regularly identify the presence of external stimuli and make decisions based on this evidence within very short time intervals (Thorpe et al. 1996; Keysers et al. 2001; Yilmaz and Meister 2013; Hoy et al. 2016). Reliable performance with limited information requires a robust representation of the external world, but the structure of neural activity that underlies representation of sensory stimuli in circumstances where evidence is fleeting or scarce is not known.

In primates and carnivores, a natural time scale exists for integrating visual information—the fixation duration. Within an intersaccadic duration (150–350 ms [Guo et al. 2006; Berg et al. 2009]; cf. ~ 1000 ms for rodents [Sriram et al. 2016]), the subject gathers information from a part of the visual scene and extracts stimulus information relevant to behavior (orientation, motion, color, etc.), suggesting that behaviorally relevant information can be extracted in a few hundred milliseconds. Rapid processing of sensory information has obvious evolutionary benefits (Yilmaz and Meister 2013; Hoy et al. 2016), but the relationship between performance and neural representation has not been carefully investigated. One reasonable hypothesis would be that animals integrate information for a duration that leads to reliable responses in cortical neurons. We sought to address this possibility by carefully comparing the reliability of cortical representation with the quality of behavioral performance.

In several species (Holmes 1918; Cowey and Stoerig 1995; Rees et al. 2002; Petruno et al. 2013), the activity of neurons in the primary visual cortex (V1) enables conscious perception of visual patterns in the world (though unconscious blind sight effects do not require V1 [Marcel 1983; Leopold 2012; Petruno et al. 2013]). Within cortical visual pathways, visual information is thought to be represented by a sparse and distributed neural code (Field 1994; Vinje and Gallant 2000; Kremkow et al. 2016). Such representations can be energetically favorable (Levy and Baxter 1996; Bell and Sejnowski 1997), have higher capacities than local codes (where each stimulus is represented by the activity of a small number of neurons), and are capable of generalization and tolerant to error. Constraining neural responses to be sparse and distributed in network models has recreated many of the properties of neurons in the early visual system (Olshausen and Field 1996). Most stimulus parameters associated with evoking sparse responses nonetheless produce reliable stimulus-locked responses. It is not known if this response reliability is required for the system to extract meaningful information. Several additional questions remain unanswered: 1) How sparse can the responses be and still have animals perform reliable discrimination? 2) What “code” do animals use to detect and discriminate between stimuli? and 3) How many neurons are required to perform these discriminations? Answers to these questions have the potential to yield insight into how animals learn, integrate, and process information over short time scales to support decision making.

In this study, we first establish that mice can rapidly integrate evidence over time to support decision making. Remarkably, we find that mice achieve plateau performance at time scales less than 100 ms. We then measure the electrophysiological responses of neurons across the layers of V1 to such short stimuli. These include neurons that send and receive inputs to various other cortical and subcortical areas (Glickfeld, Andermann, et al. 2013) and could be involved in integrating relevant visual information. We find that there is only a marginal change in V1 neural activity under these conditions and a majority of neurons show no stimulus-evoked activity even for stimuli in their receptive fields—the average V1 neuron fails to fire a single action potential on a vast majority of the trials.

To quantify how well individual neurons perform in discriminating visual stimuli, we developed a simple logistic regression model to quantify the contribution of each neuron to the discrimination task. For short-duration stimuli, we find that the vast majority of recorded units were poor discriminators with only a small fraction (~11%) consistently discriminating the orientation of the stimulus above chance. While consistent, these neurons never improved discrimination of the visual stimulus more than a few percentage above chance. Based on the model, we can project the population requirement for the orientation discrimination task. We find that mice would need to integrate from a few tens to a few hundred neurons from individually unreliable responses to account for the reliable performance of mice in the orientation discrimination task. This constitutes a small fraction (<0.1%) of the total number of neurons available to encode the stimulus in V1 of the mouse (Schuz and Palm 1989), indicating that mice can use sparse and highly unreliable neural responses to efficiently extract information to enable decision making at the limits of sensory perception.

## Materials and Methods

All procedures were performed with the approval and guidance of the Institutional Animal Care and Use Committee at the University of California, San Diego, CA and at Biogen, Cambridge, MA. We used *N* = 39 adult male and female mice for this study.

### Behavioral Training

Behavioral training methods were adapted from training systems developed previously for rats (Meier et al. 2011). Water-restricted adult (>P90) male and female mice were trained to use an operant conditioning chamber to receive water rewards while performing visually guided tasks. Water restriction and behavioral training/testing continued 5 days a week followed by weekends where subjects received water ad libitum. Measuring subject weight over time enabled careful monitoring of dehydration status. Subject weight was kept above 90% of adult, non–water-restricted weight. The operant conditioning chamber was a transparent arena with 3 ports (spaced 10 cm apart) to record mouse responses and provide water rewards. The arena was adjacent to an LCD screen (Viewsonic V3D245, 60 Hz) that displayed visual stimuli. The LCD was linearized with a Spyder2 Pro (DataColor) with a measured maximum luminance of 100 cd/m^2^ and an approximately equal luminance across the R, B, and G channels. The screen subtended an angle of 100° × 65° (width × height) with respect to the subject. Mice licked the request port (center port) to display a visual stimulus on the monitor. Mice responded to the displayed stimulus by licking one of the response ports (left and right ports). Correct responses were rewarded with a small droplet of water (~10 μL), while incorrect responses were punished with a timeout (5–20 s). Auditory feedback consisting of beeps of various frequencies and white noise stimuli were also included to further indicate the nature of the responses: correct, incorrect, try again, etc. Throughout the training and testing process, we varied the reward size and the timeout duration to maintain high motivation in the subject.

### Task Sequence and Parameters of Stimuli for Behavior

Mice learned visual discrimination tasks over many weeks performing hundreds of trials a day and many thousand trials over the course of the experiment. High performance in the orientation discrimination tasks was achieved by taking the mice through a series of shaping steps. These steps trained naïve mice in using the operant chamber effectively and in learning the structure of a self-directed 2-alternative forced-choice (2AFC) trial before training them on orientation discrimination. We describe the shaping steps for 2 experiments relating to behavior: basic characterization of orientation tuning (see Supplementary Table 1) and measuring integration times (see Supplementary Table 2). Preliminary experiments determined the specific parameters used to probe behavior in the orientation discrimination task. The spatial frequency of the gratings used for these experiments (0.08 cpd) maximized performance in most subjects. We used gratings tilted 45° to the vertical instead of vertical and horizontal gratings to prevent subtle variations in contrasts while rendering vertical versus horizontal gratings from influencing the behavior in an orientation-independent fashion. While preliminary experiments used full-screen stimuli, all behavioral data shown in the paper used a circular aperture with a diameter of ~ 60°. This ensured that the spatial frequency of the stimulus at the edge of the aperture was no greater than 0.11 cpd. This spatial frequency was not so different as to change the overall performance of the animal and yet was high enough to allow multiple cycles of gratings within the aperture such that the mean luminance presented varied by less than 2% at different times during the trial (for drifting gratings) or for different trials (for flashed gratings). When different contrasts were shown on the screen, the stimuli presented were isoluminant with the mean luminance equal to the background luminance. While identifying the integration duration of subjects, we used flashed gratings with random spatial phases instead of drifting gratings because, for the durations tested, the stimulus would have changed only a little and would have changed inconsistently from trial to trial. The randomized phases prevented the animal from using luminance information to perform the task—they had to use the overall angle of the grating presented to perform the task.

### Analysis of Behavior

We programmatically capture various facts about each trial performed by subjects. This allowed us to perform a quantitative assessment of behavioral performance. In our tasks, the subjects have complete control over when trials are requested and when they respond to trial requests. For analysis, we excluded trials that were completed within 50 ms. Such fast responses would have required unattainable motor speeds and likely indicated water-clogged ports due to incomplete reward consumption. This condition excluded <0.1% of the trials. We further excluded trials that took >5 s. This was to ensure that we only counted trials where subjects were highly motivated to perform the task, excluding trials where subjects were distracted or in a low motivational state. The fraction of trials rejected due to large Reaction times (RTs) did not exceed 3.05% in any of our subjects and averaged 1.18 ± 0.91% (mean ± standard deviation [SD]). We measured confidence intervals (CIs) on the performance (number of trials correct/number of trials in total) using the Clopper–Pearson method (Clopper and Pearson 1934) and the significance of difference in binomial proportion using Agresti–Caffo statistics (Agresti and Caffo 2000).

### Psychometric Data Fitting

**Figure 1:**
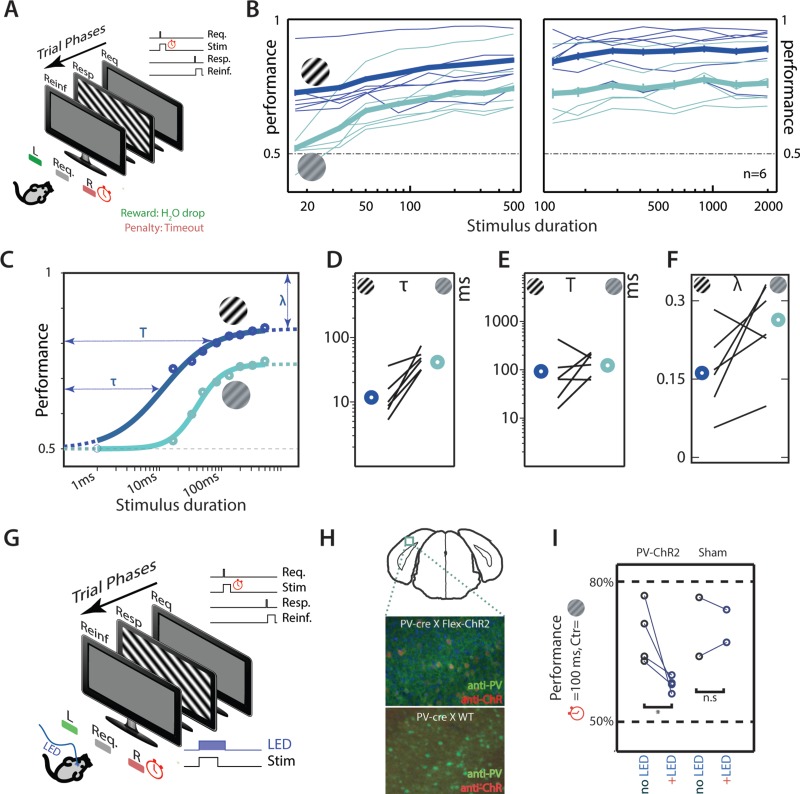
Mice integrate visual information quickly using activity in V1. (*A*) Schematic of trial structure for measuring integration time. Subjects control stimulus onset, but stimulus offset is under experimental control. (*B*) Performance improves with stimulus duration. Performance of individual subjects (thin lines) at high contrast (100%, dark blue) and at low contrast (15%, light green) as well as performance with 95% CI of average subject (thick lines) at high and low contrasts (light green) are plotted. CIs for short-duration stimuli are small and are hardly visible. (*C*) Sigmoid fits to performance of average subject at high contrast (dark blue) and at low contrast (light green). The threshold integration time (τ), total integration time (T), and lapse rates (λ) for fits obtained for high-contrast stimuli are graphically denoted. Maximal likelihood estimates of (*D*) threshold integration time (τ), (*E*) total integration time (T), and (*F*) lapse rates (λ) for the individual subjects along with the average animal (bold circles) at high contrast (dark blue) and low contrast (light green) are shown. (*G*) Schematic of behavior. Subjects control stimulus onset; stimulus offset is under experimenter control. Stimuli last 100 ms and showed low-contrast gratings (*c* = 0.15). On random half of trials, a blue LED light is delivered to fiber-optic cannulae attached to the skull of the animal. LED stimuli last 100 ms longer than the visual stimulus (*H*) Epifluorescent image of visual cortical neurons with PV+ neurons in green and neurons expressing ChR2 in red imaged from coronal slices approximately over V1 (top panel) for mice expressing ChR2 (middle panel) and sham mice (bottom panel). (*I*) Average performance in trials with (“+LED”) and without (“no LED”) LED activation for 4 subjects expressing ChR2 in PV+ interneurons and 2 sham subjects not expressing Channelrhodopsin. Average performance is significantly reduced for PV-ChR animals (paired *t*-test, *P* = 0.0345) but not for sham animals (paired *t*-test, *P* > 0.05). Abbreviations: Req.: Request; Stim: Stimulus; Resp.: Response; Reinf.: Reinforcement.

In the experiments where we vary various features of the sensory input (contrast and duration), we model subject performance as a function of the strength of the stimulus:

$$P(s)=\mathcal{F}\left(\frac{s-m}{\omega };\lambda, \gamma \right)$$

where *P* is the psychophysical performance function and $\mathcal{F}$ is the logistic function, such that

$$\mathcal{F}\left(x;\lambda, \gamma \right)=\gamma +\left(1-\lambda -\gamma \right)\left(\frac{1}{1+{e}^{-x}}\right)$$

where *s* is the strength of stimulus, *m* is the stimulus strength at half-maximum performance, ω is the width of the psychometric function (stimulus strengths where the psychometric function changes), λ is the lapse rate, and γ is the guessing rate. In our analysis of contrast threshold, we define stimulus contrast at half-maximum performance as the threshold contrast (denoted as υ), and in our analysis of integration times, we define the stimulus duration at half-maximum performance as the threshold integration time (denoted as τ). Since all our behavioral data were obtained with a 2AFC trial structure, we set γ to 0.5 in our analyses. Consistent with this assumption, when subjects were presented with trials having little or no information about the correct response (zero-contrast stimulus or very-low–duration stimulus), subjects’ performance was no better than chance. We then identified those parameters (*m*, ω, λ) that best fit our data. To identify these parameters, we used constrained maximum likelihood techniques—techniques that maximize the likelihood of the given data for some estimates of the unknown parameters, subject to constraints—to create point estimates. These analyses forced guessing rates to 0.5 (50%) and assumed that priors for the lapse rates (λ) followed a beta distribution with shape parameters (1.2, 12) while the priors for *m* and ω were flat through the stimulus range. We sampled from the posterior distribution using Markov chain Monte Carlo techniques and used the 95-percentile range of the marginal of the posterior distribution of the fitted parameters as the CI. Our analyses are based on previously described open-source methods (psignifit) (Fründ et al. 2011). In experiments where we varied the duration of the stimulus, we measured the total time required to reach 90% of the maximum performance as the total integration time (“T” in Fig. 1*C*).

### Animal Variability and Use of Average Subject

The perceptual thresholds measured varied from animal to animal. We fit psychometric tuning curves to the responses of individual animals and report the ranges of the fit parameters where applicable. When we report aggregate values, we used the values fit from the responses of an average subject. We simulated the average subject from the responses of all the animals in the population. However, different subjects performed different numbers of trials based on individual trial rates. Eight subjects were included in the contrast threshold estimation study where subjects performed between 2803 and 6078 trials averaging 4255 ± 1192 (mean ± SD). Six subjects were included in the visual integration study where subjects performed between 16 098 and 54 654 trials averaging 32 051 ± 13 896 (mean ± SD). To ensure equal weight for each animal in measuring aggregate fit parameters, we sampled the same number of trials from each subject randomly across stimulus conditions. We created the average subject by concatenating these sampled responses. To ensure that the sampling process did not bias estimates, we performed the resampling process 1000 times. We report the mean of the most likely estimates across these 1000 resamples for the average subject.

### Recording Electrode Implantation Surgery for Chronic Probes

We used standard surgical techniques to implant NeuroNexus probes (Neuronexus Inc., Ann Arbor, MI, USA) into V1. Adult mice were anesthetized under isoflurane (2.5% [v/v] for induction and 1.5% [v/v] for maintenance). After subjects were anesthetized, the fur from the top of the head was shaved and the mouse was injected with atropine to minimize secretion (0.3 mg/kg) and dexamethasone to prevent inflammation (2.5 mg/kg). Subjects were then placed on a stereotaxic frame (Stoelting Co., IL, USA). The scalp over V1 was removed using surgical scissors, and the skull dried. A small (~0.5-mm) craniotomy was made over the monocular region of V1, and NeuroNexus probes were inserted (Poly2, Poly3, and A4x2-tet configurations) into the craniotomy. The open craniotomy was covered with silicone gel. A second adjacent craniotomy was made over the olfactory bulb, and ground/reference screws were installed here. The exposed skull surface was then closed using dental cement. A custom-designed head bar was also installed to enable head fixing for later experiments. Subjects were then removed from the stereotaxic frame and allowed to recuperate in a heated chamber and injected with buprenorphine for postoperative pain management (Subcutaneous injection, 0.3 mg/kg). All V1 recordings began at least 5 days postsurgery. In a small number of animals, chronic NeuroNexus probes were installed along with movable electrode drives to sample neural populations at different depths for each session.

### Headcap Surgery for Acute Recording Preparation

The surgical procedure was identical to that of the chronic probe surgery in all respects except that no craniotomy was performed over V1. The skull surface was cleaned, and the area over V1 was covered with a thin layer of cyanoacrylate-based glue and allowed to dry completely. The animal was allowed to recover for 5 days after surgery.

### Channelrhodopsin Expression and Fiber-optic Cannula Implantation

Channelrhodopsin-2 (ChR2) was targeted into parvalbumin-positive (PV+) fast-spiking interneurons within V1 through 1 of 2 ways: 1) PV-cre (JAX:0080609) subjects were injected with 200–400 nL of AAV2-Flex-ChR2-tdTomato (UPenn Vector core Cat# AV-9-20297P) at various depths using a Nanoject II (Drummond Scientific) bilaterally over V1, and 2) PV-cre animals (JAX:0080609) were crossed with Flex-ChR2 (JAX:024109) and F1 progeny positive for both genes were selected for behavior. Fiber-optic cannulae (MFC_480/500-0.63_3mm_ZF1.25(G)_B60, Doric Lenses Inc.) were implanted into the open craniotomies over V1 such that the fiber terminus lay over the exposed brain surface. The open skull was covered with dental cement, and the mouse was removed from the stereotaxic frame and set aside for recuperation. As a light stimulation negative control, 2 subjects underwent a sham injection. These subjects (PV-cre:0080609) were implanted with fiber-optic cannulae over V1 without viral injection.

### Optogenetic Manipulation During Behavior

Subjects implanted with fiber-optic cannulae were allowed to recuperate for 5 days, after which they were reintroduced into the behavior chamber. We established baseline behavioral performance in the orientation discrimination task for ~ 1 week before attaching the animal to a fiber-optic light source. Transistor-Transistor Logic (TTL) pulses from the behavior computer controlled the LED light source (M470F3, Thorlabs) through an LED driver (LEDDB1, Thorlabs). This light entered the behavior arena via an optical commutator (FRJ_1x1_FC_FC, Doric Lenses), then split into two through a 1 × 2 branching fiber-optic patch cord (BFP(2)_480/500/900-0.63_0.3m_FCM-2xZF1.25(F), Doric Lenses), and mated with the cannulae with a plastic sleeve. Based on prior estimates of stimulus latency within V1 (Haider et al. 2013), we extended the LED light stimulation past the visual stimulus by 100 ms. Thus, LED lights started with stimulus onset and finished ~ 100 ms after stimulus offset. We estimate the LED power to be ~ 9 mW at the fiber tip leading to a power density of ~ 12 mW/mm^2^.

### Stimulus Presentation and Electrophysiological Recording from Chronic Probes

Subjects that underwent electrode implantation surgery were allowed to recuperate for 5 days. We acclimated subjects to being head fixed for 2 days before recording from V1 neurons. Subjects were head fixed by screwing the custom-designed head bar to a mating bar. The mating bar was then attached to the recording rig with the mouse placed over a Styrofoam ball suspended in air to allow free range of motion (Dombeck et al. 2007; Niell and Stryker 2010) or over a vertical wheel capable of rotating along a horizontal axis (Ramesh et al. 2018). While the mouse was head fixed, an LCD monitor (Viewsonic V3D245) was placed contralateral to the electrode implantation site. The LCD was the same distance away from the head-fixed subject as it would have been during behavior and was linearized in an identical fashion. The location of the monitor was adjusted to drive visual responses in the V1 neurons being recorded. The raw waveforms from V1 were buffered, filtered, and digitized to a hard drive using the Open Ephys (Siegle et al. 2015) or OmniPlex (Plexon Inc.) system. Coincident with the physiological recording, we recorded synchronizing TTL pulses from the display computer to align spikes with the stimulus. Stimuli presented to the subject were similar in characteristics to the stimuli used to drive behavior, except for a few characteristics. To maximize the number of neurons that were driven by stimuli, we chose to use full-screen stimuli instead of through a circular window. An initial recording epoch collected the responses of subjects to gratings of different orientations (full contrast, 12 orientations, flashed for 500 ms or drifting for 2000 ms). The responses to these stimuli were used to characterize the orientation tuning of the neurons. Some sessions included responses to long-duration stimuli (2000 ms) of full-contrast (100%) and low-contrast (15%) drifting gratings (temporal frequency of 2 Hz) tilted 45° from the vertical. After this characterization, we recorded responses of neurons to flashed gratings of short durations (50–200 ms). These included trials where the contrast presented was zero and no stimulus was shown on the screen. These trials measured background firing rates for the neuron. In a small number of sessions, we performed pupillometry as well as measured the running speed of the subject as a measure of arousal (Bradley et al. 2008; Niell and Stryker 2010; Reimer et al. 2014).

### Pupillometry and Pedometry

In sessions where pupillometry was performed, videos of the mice’s pupils were captured with a Stingray camera with infrared (IR) filter removed while recording the mouse’s eyes under IR illumination (850 nm) using a Computar MLH-10X zoom lens. Visible light was excluded from the camera using a spectral filter (LP800-46, Midwest Optical Systems Inc.). Eye tracking was synchronized with neural recording using the Cineplex system (Plexon Inc., TX, USA). To measure pupil size from the video, a deep learning algorithm was trained on sample eye-tracking videos from multiple subjects across multiple days (Deeplabcut 2.0 [Mathis et al. 2018]). Based on this algorithm, the major and minor axes of the pupil were identified across all the data along with the location of the corneal reflection (Fig. 5*C*), and this measurement was used to measure the size of the pupil. Measured sizes were smoothed with a median filter (1 s) and *z*-scored to identify epochs of high engagement (*z* > 0) and low engagement (*z* ≤ 0). In these animals, running speed was measured using an optical motion sensor (PWM3360) placed against the outer surface of the wheel. Motion data were captured by an Arduino device programmed with custom code, converted to analog voltages (MCP4725 digital–analog converter, Adafruit industries), and synchronized with the neurophysiological recording using the Omniplex System (Plexon Inc.). In our hands, epochs of high locomotion coincided with epochs of larger pupil sizes. Thus, we used pupil size as the sole measure of arousal.

### Stimulus Presentation and Electrophysiological Recording from Acute Probes

Subjects were allowed to recover for 5 days postsurgery and were acclimated to the Styrofoam ball for at least 2 days before the recording. On the day of the recording, subjects were lightly anesthetized, a small craniotomy was performed over V1, and the exposed brain was covered with silicone gel. The animal recovered from anesthesia for at least 2 h before recording.

### Single-Unit Identification

Raw neural data were filtered between 300 and 10 000 Hz using a zero-phase digital filter. Open source libraries (Rossant et al. 2016) (spikedetekt) were used to detect putative spikes as significant voltage deviations (5σ strong threshold, 2.5σ weak threshold). Detected spikes were automatically clustered using an expectation maximization algorithm (klusta [Rossant et al. 2016]), which modeled features (principal components of waveforms) of neurons as a mixture of Gaussians. Clustered single units were manually verified using a visualization algorithm (kwik-gui). Overclustered units were combined based on the location of detected spike on the electrode, waveform shape, feature stability, and absence of refractory violations. Single units showed no refractory violation and were sufficiently separated from other units such that total false-positive + false-negative rates are less than 5% (Hill et al. 2011). For each unit, we extracted the location within the brain calculated as the location of the electrode that had the largest mean waveform amplitude. This depth was used to categorize the unit as belonging to the superficial (<400 μm, Layer 2/3 [L2/3]) or deep (>450 μm, L5/6) layers of the cortex. As we were recording with chronic electrodes, some units were detected on the same electrodes across multiple days. These units were identified by looking for units with 1) the same waveform shape (Pearson correlation > 0.95 across days) and 2) the same interspike interval (ISI) distribution (Pearson correlation > 0.95 across days) present at 3) the same depth across days. We found 190 V1 units that were present across multiple days. Duplicate units were removed such that only the responses of the units on the first day they were present were considered for future analysis.

### Analyzing V1 Responses

We synchronized spiking responses in V1 with stimulus presentation using TTL pulses. Unless otherwise specified, on each stimulus presentation, we extracted the total number of spikes in a time window that started with the visual stimulus onset and extended to 500 ms after onset. We chose to analyze this time interval for 3 reasons. 1) V1 responses do not begin immediately after stimulus onset. Capacitive charging effects and line delays cause a response latency of ~ 50–100 ms (Gao et al. 2010; Haider et al. 2013). 2) V1 responses could sometimes extend far beyond the duration of the stimulus due to recurrent activity within the network (Reinhold et al. 2015). 3) Analysis of various time intervals showed that a spike number code that included the chosen time interval (0–500 ms) maximized the average decoding performance across all neurons (Fig. 3*B*), had close to the maximum number of consistent predictors of the stimulus (Fig. 3*B*), and completely covered the stimulus in all conditions.

### Fitting Performance of Individual Neurons and for Populations of Neurons in a Session

Spike rates for each neuron were considered one at a time. We first excluded the firing rates for trials without stimuli (i.e., contrast = 0). The firing rates for the remaining trials were randomly assigned to “Training” (70%) and “Test” (30%). The data in the Training set were used to fit a logistic regression model (statsmodels v0.9.0 in Python 3.6) and logistic regression coefficients obtained for each neuron. This regression coefficient was used to test the model on the Test dataset (along with the no-stimulus trials). We repeated this process 100 times with a different subset of trials belonging to the Training and Test datasets. The results of regression included the probability that the extracted coefficient was significantly different than 0. If this probability was high (>95%) in at least 70 out of 100 different splits, the unit was considered a consistent predictor (i.e., they predict the stimulus as belonging to the same orientation no matter which trials are included in the fitting process). For fitting performance across populations, we used all the spike rates in a session and performed the regression in a similar fashion. The decoding performance of a single unit or a population was the average performance across the 100 splits.

### Simulating Neural Subpopulations and Measuring Performance of Simulated Populations

For each of the neurons in our dataset, we tabulated the spike count responses according to the stimulus conditions tested in our study (contrasts of 0, 0.15, and 1 and durations from 50 to 200 ms). To create a virtual session of a given population size from our dataset, we first chose a random subset of that size from the overall population with replacement with each neuron being equally likely to be included in the subpopulation. Responses for each neuron were then simulated from the corresponding response table making sure to only sample from the responses of that neuron for that stimulus condition. We used the previously calculated regression coefficients (calculated one neuron at a time) as the regression coefficient for the simulated neuron. The orientation of the stimulus was then predicted based on these independent regressors and compared against the input orientation. This was repeated 1000 times to provide an estimate of the performance of a population of that size.

## Results

### Mice Performing Orientation Discrimination Task Integrate Information Over Very Short Time Scales

First, we trained adult mice in an orientation discrimination task. Naïve mice were introduced into the training arena with 3 response ports and their behavior slowly shaped to the appropriate response contingency (see Supplementary Figure 1A; see also Behavioral Training and Task Sequence and Parameters of Stimuli for Behavior). We captured the contrast dependence of the orientation discrimination task in a series of trials where we varied the contrast of the discriminandum. As expected, we find that all subjects improved performance with increasing contrast (see Supplementary Figure 1B, gray lines, performance at *c* = 1.0 greater than performance at *c* = 0.15 for 8/8 mice). We fit the performance of mice across contrasts to a logistic regression curve (see Supplementary Figure 1B, C and Supplementary Results, contrast tuning of orientation discrimination) and identified the threshold contrast to be *c* = 0.15. Furthermore, we found that the animal’s reaction time was shorter for higher contrast stimuli (see Supplementary Figure 1E) and performance of the animal increased the longer it looked at the stimulus (see Supplementary Figure 1F and Supplementary Results, integrating information in orientation discrimination task across contrasts), indicating that the mice can integrate evidence.

To precisely measure the time window over which mice effectively integrate information, we tested subjects trained in orientation discrimination to perform trials where the maximum duration of a stimulus available for the subject is systematically controlled (see Task Sequence and Parameters of Stimuli for Behavior). After this maximum duration (denoted “stimulus duration”), the screen changes to a gray screen and awaits response from the subject (Fig. 1*A*). This trial structure puts limits on how long subjects can integrate visual information—stimuli after the stimulus duration do not contain useful information to perform the task. All subjects improved performance as the stimulus duration increased (Fig. 1*B*, thin lines, performance at 500 ms > 16 ms in all animals, *P* < 0.05, Agresti–Caffo statistics). This was true for high-contrast stimuli (Fig. 1*B*, blue lines) as well as lower contrast stimuli (Fig. 1*B*, green lines). For example, while no subject had performance significantly above chance for a 16-ms stimulus at low contrast (*c* = 0.15), all subjects perform significantly above chance for stimuli lasting 50 ms at low contrast. At higher contrast (*c* = 1), all subjects were significantly above chance for a stimulus duration of 16 ms, the shortest stimulus duration tested. To precisely measure the dynamics of integration, we fit a logistic regression curve to the stimulus duration versus performance curve (see Psychometric Data Fitting and Animal Variability and Use of Average Subject; Fig. 1*C*). This allowed us to measure the threshold integration time (τ), total integration time (T), and lapse rate (λ) (see Psychometric Data Fitting for definitions). Based on these fits, individual mice had a threshold integration time between 13 and 46 ms at high contrast (*c* = 1) averaging 24 ± 12 ms (mean ± SD; Fig. 1*D*) and a threshold integration time between 33 and 75 ms at low contrast (*c* = 0.15) averaging 54 ± 16 ms (mean ± SD; Fig. 1*D*). Thus, subjects require very short stimulus durations—significantly shorter than the typical intersaccade duration—to perform significantly above chance in an orientation discrimination task. The average subject had a threshold integration time of 12 ms at high contrast (Fig. 1*D*, blue circle) and 45 ms at low contrast (Fig. 1*D*, green circle), an order of magnitude lower than the mean reaction times (~1 s) from the reaction time task described earlier.

Our assessment of the total integration time indicated that it was between 20 and 400 ms for high-contrast stimuli and between 55 and 250 ms for low-contrast stimuli (Fig. 1*E*). The total integration time for the average subject was 98 ms at high contrast and 107 ms at low contrast (Fig. 1*E*). This duration is an order of magnitude smaller than mean reaction times. The lapse rates for high-contrast stimuli (0.33 ± 0.15) (Fig. 1*F*) were consistent with the lapse rates of subjects at high contrasts (0.31 ± 0.14) in the reaction time task (see Supplementary Figure 1C; *P* = 0.85, Mann–Whitney–Wilcoxon *U* test). Similarly, plateau performance for low-contrast stimuli (mean ± SD = 0.74 ± 0.08) is comparable with the performance of subjects for low-contrast stimuli (mean ± SD = 0.67 ± 0.1) in the reaction time task (*P* = 0.18, Mann–Whitney–Wilcoxon *U* test). This indicates that subjects have integrated as much of the information from the visual stimulus as possible within ~ 100 ms to guide their behavior.

### Activity in V1 Is Necessary for Performing the Orientation Discrimination Task

To test if V1 neurons are required for orientation discrimination, we expressed blue-light–sensitive ChR2 in PV+ inhibitory interneurons (Fig. 1*H*, middle panel) in V1, which should suppress activity of projection neurons in V1 when activated. Suppression of cortical activity by the activation of PV+ interneurons is known to be immediate and reversible (Lien and Scanziani 2013; Reinhold et al. 2015). We tested the causal role of V1 activity in behaving animals by activating Channelrhodopsin on random trials while the subjects performed an orientation discrimination task (Fig. 1*G*; also see Optogenetic Manipulation During Behavior). Performance in trials where ChR2 was activated was significantly lower than in trials where no ChR2 activation occurred (Fig. 1*I*, PV-ChR2 no LED vs. +LED; *P* < 0.05, Mann–Whitney–Wilcoxon *U* test). We confirmed that the loss in performance was not due to the distracting influence of the blue light by performing identical experiments in a small cohort of sham animals (*n* = 2) that did not express ChR2 in PV+ inhibitory interneurons (Fig. 3*E*, bottom panel). While the small sample size does not allow us to be certain of the effects of the distracting effects of blue light in these sham animals, performance in light-activated trials was no different than the performance in trials without light activation (Fig. 1*I*, sham/no LED vs. +LED; not significant, Mann–Whitney–Wilcoxon *U* test). Thus, activity of neurons in V1 is necessary for mice to carry out this orientation discrimination task.

### Activity of V1 Neurons Is Sparse and Unreliable

**Figure 2:**
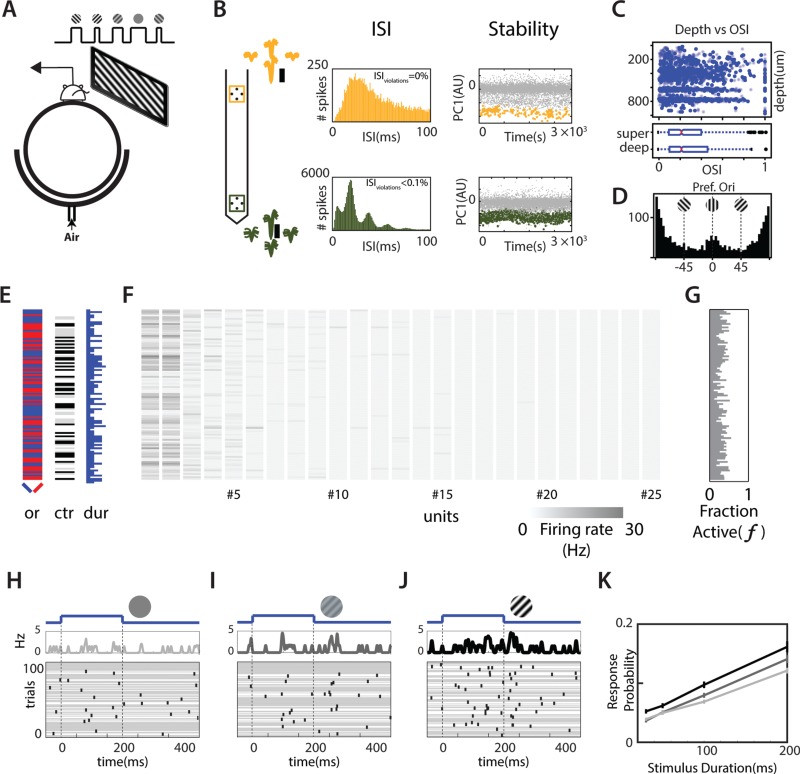
Statistics of neural responses in V1. (*A*) Schematic of recording setup. Subjects run on a Styrofoam ball suspended in air while recording in the left V1. We show visual stimulation to the right eye on an LCD monitor placed ~ 15 cm from the eye, tangential to the eye. (Top panel) Stimuli are gratings of different orientations, contrasts, and durations separated by short periods (~1 s) of gray screen. (*B*) Waveform, ISI, and first principal component of one single unit (top, yellow) and one multiunit (bottom, green) simultaneously recorded in V1 on 2 separate tetrodes. (*C*) Distributions of OSI across the population as a function of depth of the unit (top panel; solid blue: single units, light blue: multi units) as well as box plots of the distributions for superficial (super) and deep units (bottom panel: box plots of OSI distributions for superficial and deep units). (*D*) Distribution of preferred orientation (Pref. Ori) calculated from the preferred orientation vectors. (*E*) The orientation (or) (left = blue, right = red), contrast (ctr) (0: white, 0.15: gray, 1: dark gray), and duration (dur) (50--200 ms) of the stimulus of the first 100 trials of a single session. (*F*) Heat map of the spike rates of simultaneously recorded neurons in a time window that started with stimulus onset and ended 500 ms after stimulus onset. Inset shows the scale of the heat map. This shows 25 out of 39 neurons recorded in this session. (*G*) The fraction of total recorded neurons that produced at least one spike in the time window of interest. (*H–J*) Raster of the responses of a single L2/3 neuron with preferred orientation to the right as a function of contrast. Stimulus orientation and contrast (image) as well as onset and offset times (blue curve) are provided above the raster. In these panels, trials without a single spike are denoted by a gray line through the duration of the trial. (*K*) The probability of neurons responding to stimuli with a single spike at different contrasts (*c* = 1, black; *c* = 0.15, dark gray; *c* = 0, light gray) as a function of duration of stimulus.

To understand the neural basis for fast visual integration, we recorded from neurons across the layers of V1 in subjects running on a Styrofoam ball or on a vertical wheel while passively viewing stimuli of various contrasts, durations, and orientations (Fig. 2*A*). Visual responses of neurons from 19 subjects across a total of 119 sessions were captured. Of these, 65 sessions (*N* = 9 mice) were recorded in naïve animals that had no exposure to the behavioral arena and 16 (*N* = 6 mice) were recorded in animals that had prior experience in the orientation discrimination task and had threshold performance in the simple orientation discrimination task (see Step 4, Supplementary Table 2). Additionally, 38 sessions from 4 subjects included simultaneous pupillometry and pedometry. Our dataset contained a total of 2373 units, of which 978 were well-isolated single units (Fig. 2*B*, top panels) and 1395 were multiunit (Fig. 2*B*, bottom panels) activities (see Supplementary Figure 2B,C; also see Single-Unit Identification). These units had firing rates that ranged from a minimum of 0.2 Hz to a maximum of 80.9 Hz (see Supplementary Figure 2D). Most neurons fired very few spikes over the course of the session (see Supplementary Figure 2D, mode at the lowest firing rate). Firing rates were dependent on the depth of the recording (superficial [L2/3]: depth < 400 μm, deep [L5/6]: depth > 400 μm, Kolmogorov-Smirnov (KS) test; *P* < 10^−3^; see Supplementary Figure 2E). Spike widths varied from 0.07 to 0.87 ms based on peak-to-trough duration (see T in Supplementary Figure 2F). Spike widths showed a bimodal distribution (see Supplementary Figure 2G, left panel). Cluster analysis using k-means clustering (similar to Niell and Stryker 2008) indicated that 2 overlapping clusters could be identified. Among well-isolated single units, the first cluster contained units with short spike widths and high firing rates (mean = 0.26 ms, 15.2 Hz), whereas the second contained units with long spike widths and lower firing rates (mean = 0.54 ms, 5.6 Hz), and these differences were significant (*P* < 0.05, Mann–Whitney–Wilcoxon *U* test for each respective comparison). We denote these as “fast-spiking” and “regular-spiking” neurons respectively. We expect that these clusters are enriched in fast-spiking, PV+ interneurons and regular-spiking excitatory neurons, respectively.

As expected from neurons in V1, a large fraction of neurons showed significant orientation tuning (Fig. 2*C*). For a subset of neurons (2206 units), we calculated orientation tuning (see Supplementary Figure 2H,I, polar plot) and vector sums of orientation tuning (see Supplementary Figure 2I, arrows) as measures of neural tuning to orientation (similar to prior techniques [Mazurek et al. 2014]) based on high-contrast flashed gratings of different orientations that lasted 500 ms or drifting gratings that lasted 2000 ms (see Supplementary Figure 2H). For all these neurons, we further calculated Jackknife error estimates of these measures by removing data from one trial at a time. Neurons showed varied selectivity and orientation preferences (Fig. 2*C*,*D*).

**Figure 3:**
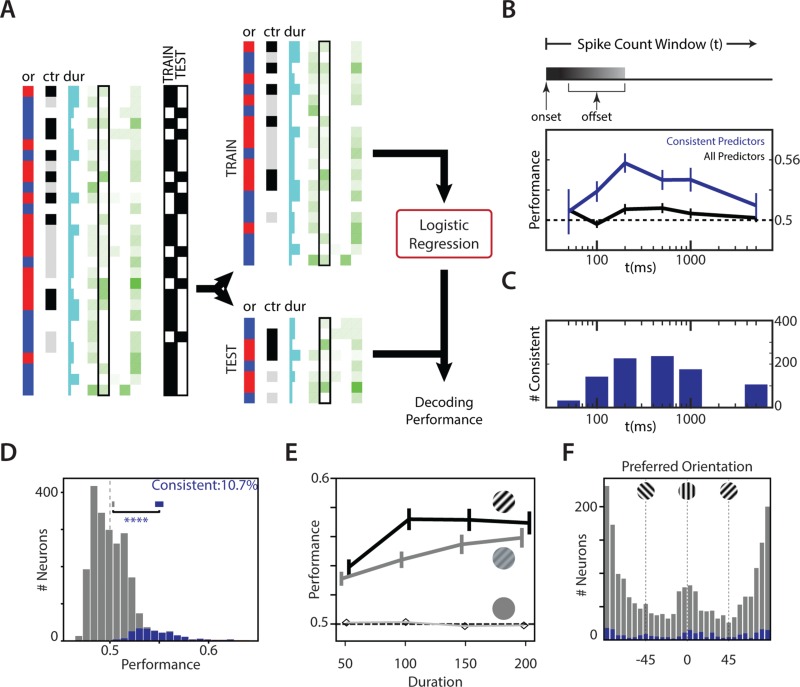
Contribution of individual neurons to orientation discrimination. (*A*) Schematic of logistic regression fit. The responses of the neuron were split into 70% training (TRAIN) and 30% testing (TEST), and spike counts were used as features for a logistic regression. Regression coefficients calculated from the TRAIN dataset were tested on the TEST dataset to obtain decoding performance. (*B*) Average decoding performance of individual neurons across the population as a function of the spike count window. Performance for each neuron was measured as the average decoding performance for that neuron across a hundred independent splits of training–testing subsets. Top panel: Spike count window begins on stimulus onset and could extend past stimulus offset. Bottom panel: the average decoding performance of all units (black) and consistent units (neurons that reliably improve the logistic fit upon inclusion no matter how the data are split; blue) as a function of the spike count window (*t*). Error bars are 95% CI of the mean. (*C*) The number of consistent predictors in the population as a function of the spike count window. (*D*) Histogram of decoding performance of units (calculated as in *A*, with spike counts over 500 ms as features) in the recorded population. A performance of 0.5 indicates no information. Mean ± 95% CI of performances of the overall population (gray) and the consistent subpopulation are also shown (****P <  0.001). (*E*) Duration and contrast dependence of the performance of neurons. Data show mean ± 95% CI performance for 15% (gray) and 100% (black) contrasts. Curves have been shifted slightly in the *x*-direction to aid in visualization. Mean performance for 0% (unfilled) is also shown without CIs. (*F*) The preferred orientation of consistent predictors (blue) compared with that of the overall population (gray). Abbreviations: or, orientation; ctr, contrast; dur, duration.

The orientation selectivity index spanned values from 0 to 1 with a mean selectivity of ~ 0.31 (Fig. 2*C*), while the preferred orientation spanned the entire orientation space with significant preference for vertical and horizontal orientations (Fig. 2*D*, *P* < 10^−4^, KS test vs. uniform null hypothesis). We note that a small population of neurons (*N* = 168) show very high orientation selectivity (Orientation Selectivity Index; OSI > 0.95). These neurons have significantly lower firing rates compared with the rest of the population (0.21 Hz for high OSI vs. 5.94 Hz for the population; *P* < 10^−4^, Mann–Whitney–Wilcoxon *U* test). The high OSI was due to an extremely low firing rate for the orthogonal orientation, but each of these neurons had consistent orientation tuning—the SD of Jackknife estimates of orientation preference was less than 20° in all high-OSI units with a median of 2.5°. Furthermore, putative regular-spiking neurons had a higher mean OSI than fast-spiking neurons (0.31 vs. 0.27; *P* < 0.05, Mann–Whitney–Wilcoxon *U* test). Thus, as shown previously, we find that neurons in V1 are orientation tuned and are ideally poised to encode the stimulus orientation to direct the animal’s behavior.

Next, we characterized the population responses of simultaneously recorded neurons to stimuli that last a short duration (≤200 ms). We recorded between 2 and 54 neurons (see Supplementary Figure 2C, top panel) simultaneously in our recording sessions. We show the stimulus (orientation, contrast, and duration) (Fig. 2*E*) spike counts in a time window that spanned the first 500 ms after stimulus onset of 25 (out of 39) simultaneously recorded neurons for the first 100 trials of one session (Fig. 2*F*). For each trial, the fraction of the recorded population that responded to the stimulus with at least one spike (Fig. 2*G*) was computed. For the session shown, the average fraction of neurons that responded to the stimulus with at least one spike was 36 ± 9% (mean ± SD). Across all sessions and all trials, the fraction of V1 neurons that responded with at least one spike in the 500-ms window was 43 ± 17% (mean ± SD). This fraction of responsive neurons varied with the contrast and duration of the stimulus used (see Supplementary Figure 2J,K). We measured the mean population fraction for each session as a function of the stimulus parameters. For stimuli that lasted 100 ms, this mean fraction was lower for zero-contrast stimuli compared with the fraction at high-contrast stimuli (see Supplementary Figure 2K; *f* = 37% at *c* = 0 vs. *f* = 43% at *c* = 0.15, and *f* = 37% at *c* = 0 vs. *f* = 44% at *c* = 1). The difference between the fraction of responsive neurons was not significantly different across any of the stimulus conditions (Mann–Whitney–Wilcoxon *U* test, adjusted for multiple comparisons). Thus, for such short stimuli, merely looking at the number of active neurons would not indicate the presence or absence of the stimulus. These results indicate that, for stimuli that drive reliable behavior, stimulus onset increases the fraction of responsive V1 neurons by at most 8%.

### Effect of Visual Stimulus on Spike Probability of V1 Neurons

Sparse responses could still underlie reliable behavior if individual neurons could respond reliably. To study the reliability of neural responses, we measured the probability that neurons would respond with at least one spike to stimulus presentation. We show the responses of one such L2/3 neuron with preferred orientation to the right of vertical (Fig. 2*H*–*J*). To assess the influence of contrast on spike probability, we calculated the fraction of trials that elicited at least one spike during the time that spanned the stimulus presentation and extended 100 ms after stimulus offset. The spike probability changed as we varied contrast at the preferred orientation (Fig. 2*H*–*J*). Trials where the neuron failed to respond with a single spike are denoted with gray lines across the duration of the trial. The neuron responds unreliably even for high-contrast stimuli (Fig. 2*J*), indicating that individual neurons may not fire reliably for stimuli that drive reliable behavior.

To quantify the contrast and duration dependence of response probability, we split the recorded population of neurons into populations sensitive to orientation tilted to the right of vertical and to the left of vertical. Across the population, even at the highest contrast and highest duration presented, neurons fire spikes on fewer than 20% of trials (Fig. 2*K*, black) on average. Responses at lower contrasts (Fig. 2*K*, gray) and durations elicit spikes on fewer trials. Thus, for the stimulus conditions that drive reliable behavior (low-contrast, 100-ms stimuli/high-contrast, 50-ms stimuli), neurons that are responsible for encoding that behavior fire fewer than 0.1 spike every trial.

### Individual Neurons Encode Short Stimuli Poorly

To understand the extent to which individual neurons might contribute to reporting the orientation of the stimulus, we used the trial-to-trial responses to decode the orientation of stimuli. We split the responses of individual sessions across differing durations, orientations, and contrasts into Training (70%) and Test (30%) sets (Fig. 3*A*; see Fitting Performance of Individual Neurons and for Populations of Neurons in a Session). A logistic regression model was created on the training set and used on the test data to obtain decoding performance for the neuron (Fig. 3*A*) as well as identify specific units that are consistent predictors of the orientation of the stimulus (see Fitting Performance of Individual Neurons and for Populations of Neurons in a Session). To identify the window over which we can best identify the orientation of the stimulus from neural responses, we performed the decoding analyses for different spike count windows (Fig. 3*B*,*C*). The average decoding performance across the population was low but peaked around 500 ms (Fig. 3*B*, black). Consistent predictors were significantly better than the overall population at decoding the orientation of the stimulus for spike windows ranging from 100 ms to 1 s (Fig. 3*B*, blue). For all further analyses, we chose to focus on the spike counts over 500 ms because it 1) completely covered all the stimulus conditions tested in our dataset (Fig. 3*B*, top panel) and 2) approximately maximized the average information in the neural responses (Fig. 3*B*, bottom panel) while 3) maximizing the number of consistent predictors (Fig. 3*C*).

**Figure 4:**
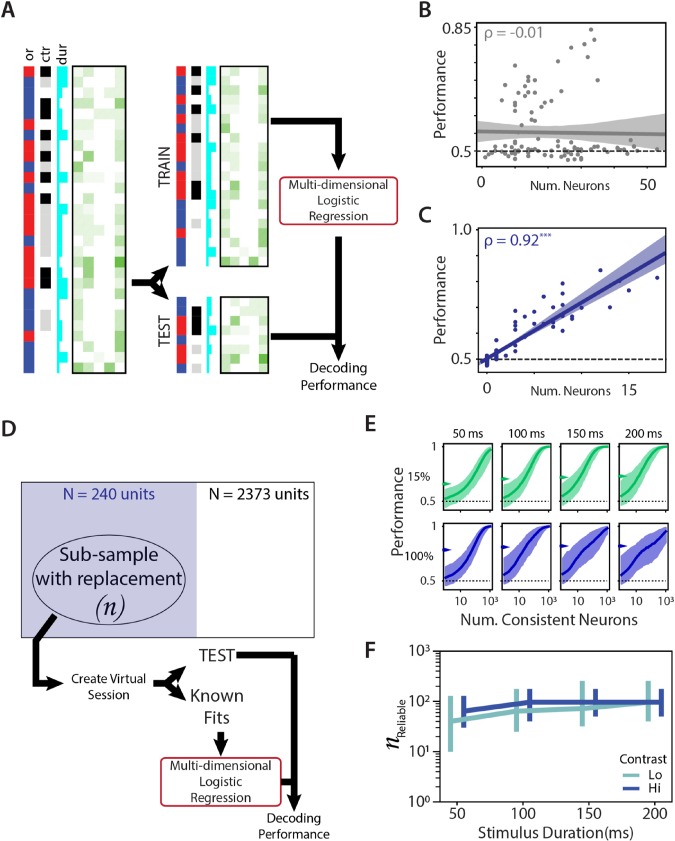
Integrating evidence across neurons improves performance. (*A*) Schematic of logistic regression fit. The responses of the neuron were split into 70% training (TRAIN) and 30% testing (TEST), and spike counts across all neurons were used as features for a multidimensional logistic regression. Regression coefficients calculated from the TRAIN dataset was tested on the TEST dataset to obtain decoding performance. Multidimensional logistic regression fits obtained from training data and tested on test data as a function of (*B*) total number of units in the session or (*C*) total number of consistent units in the session. Circles indicate performance of individual sessions. Solid line indicates best linear fit, and shaded area indicates the CI of the linear fit. (*D*) Schematic of resampling method to create virtual sessions of different subpopulations of neurons. “Virtual” neurons were chosen with replacement from the set of consistent neurons (blue), and spike counts of these neurons simulated by sampling from their respective response distribution. Prior logistic fits were then used to estimate performance. (*E*) Relationship between number of consistent neurons and performance measured for virtual sessions created as in *D* for gratings of low (15%, green, top row) or high (100%, blue, bottom row) contrast that lasted between 50 and 200 ms (left to right columns). Solid lines indicate median performance, and shaded areas indicate 95 percentiles of performances across 1000 subsamples. Arrows indicate the measured behavior for the average subject for the specific stimulus. (*F*) Number of reliable neurons required to perform as well as an average subject as a function of stimulus duration for high contrast (blue) and low contrast (green). Data show median ± 95 percentiles. Abbreviations: ctr, contrast; dur, duration; Hi, high; Lo, low; Num., number; or, orientation.

Individual neurons encoded the orientation of the stimulus poorly. The performance of individual neurons across all stimulus conditions varied from 0.46 to 0.64 (Fig. 3*D*), compared with chance performance of 0.50. The performance of consistent predictors was marginally but significantly higher than the overall population (Fig. 3*D*, blue; 0.55 vs. 0.5, *P* < 10^−6^, Mann–Whitney–Wilcoxon *U* test). This subpopulation 1) was enriched in single units compared with the overall population (49% vs. 41%, Agresti–Caffo statistics, *P* < 0.05), 2) included units with firing rates from 0.1 to > 80 Hz (see Supplementary Figure 3A), 3) showed increases in performance with increased contrasts and durations in a manner consistent with the improvement in performance of subjects in the orientation discrimination task (cf. Fig. 3*E* vs. Fig. 1*B*), and 4) had units whose orientation preference was enriched in angles around the discriminated stimulus (Fig. 3*F*). Specifically, the fraction of units with preferred orientations within 10° of the discriminated stimuli was higher in the consistent subpopulation (0.16 vs. 0.1, *P* = 0.023, Agresti–Caffo statistics), and the overall distribution of orientation preference was significantly different between the consistent and overall populations (KS test, *P* = 0.004). Consistent units included both regular- and fast-spiking units in approximately the same proportion that they were present in the overall population. Both categories discriminated the stimulus to the same degree (Mann–Whitney–Wilcoxon *U* test, *P* > 0.05).

Responses to longer duration stimuli (±45°, contrast = 1 and 0.15, drifting at 2 Hz for 2 s) were captured in a subset (781 out of 2373) of units (see Supplementary Figure 3B). Identical decoding analyses of the orientation of the stimulus from this subset revealed that 1) a greater fraction of neurons were consistent predictors of the orientation of the stimulus (35.4% vs. 10.7% for short-duration stimuli, *P* < 0.001, Agresti–Caffo statistics), 2) the average performance of the consistent population was significantly higher (0.61 vs. 0.55, *P* < 10^−4^, Mann–Whitney–Wilcoxon *U* test), and 3) a small population of units (10/781) performed better than the performance of the animal (see Supplementary Figure 3C). Decoding performance from responses to long- and short-duration stimuli were highly correlated (Pearson ρ = 0.45, *P* <   0.001; see Supplementary Figure 3C), indicating that the responses to long-duration stimuli were qualitatively similar to but quantitatively different than responses to minimally discriminable stimuli. Nevertheless, longer duration stimuli can drive reliable responses such that the activity of one or a few neurons is sufficient to decode the orientation of the stimulus. In contrast, for minimally discriminable short-duration stimuli, even the best performing individual neuron was not close to the performance of the whole animal (e.g., ~ 80% behavioral performance for full-contrast stimuli that lasted 100 ms or ~ 70% for low-contrast stimuli that lasted 100 ms), indicating that any strategy that relies on the response of an individual high-performing neuron was unlikely to drive the behavior of the animal.

### Integrating Information Across Neurons Predicts Orientation

Since even the best performing single units were poor predictors of stimulus orientation, we next explored how integration across relatively unreliable units might affect stimulus prediction. To determine how decoding of stimulus improves with integration of information from multiple neurons, we used the simultaneously recorded spike rates in all the neurons recorded in a session to fit a multidimensional logistic regression similar to the method employed for the single neuron fits (Fig. 4*A*; also see Fitting Performance of Individual Neurons and for Populations of Neurons in a Session). While performance with multidimensional logistic regression was not directly correlated with the number of simultaneously recorded neurons in the session (Fig. 4*B*; ρ = −0.01, *P* = 0.81), it correlated well with the number of consistent neurons recorded in the session (Fig. 4*C*; ρ = 0.92, *P* < 10^−3^). There was a rapid increase in stimulus prediction performance with recruitment of additional neurons indicating a high sensitivity to integrating information from even a relatively small number of neurons.

To estimate the number of neurons required to perform as well as the animal, we simulated new sessions using increasing subpopulation of neurons from the overall population. Each neuron in the subpopulation was selected at random with replacement from the original population and firing rates for multiple trials simulated from known responses of the neuron for similar stimuli (Fig. 4*D*; also see Simulating Neural Subpopulations and Measuring Performance of Simulated Populations). We used these virtual sessions along with known logistic fits for individual neurons to create a multidimensional logistic regression (Fig. 4*D*). This analysis revealed that performance improved with the number of neurons and was dependent on the contrast (Fig. 4*E*, top row vs. bottom row) and duration (Fig. 4*E*, left to right columns) of the stimulus. Consistent with the absence of visual information at zero contrast, the decoding performance of populations of neurons to zero-contrast stimuli was at chance level (see Supplementary Figure 4A). We used the population dependence curves along with known performance of the average subject (Fig. 4*E*, arrows) in the behavioral task to estimate how many consistent neurons would be required to decode the stimulus as reliably as the average subject could. Across stimulus conditions, a few tens to a few hundred neurons are required to match the performance of subjects from behavioral experiments (Fig. 4*F*), averaging a median of 68 neurons for low-contrast stimuli and 88 neurons for high-contrast stimuli. This still represented < 0.1% of the neurons in V1 and suggests that information contained in the sparse and variable firing pattern in V1 is sufficient to account for visually guided behavior.

## Discussion

Our assessment of the time scale of evidence integration for visually guided decision making was enabled by the development of an automated platform for studying visual function in rodents (Meier et al. 2011). We adapted this platform for mice such that dozens of mice would perform hundreds of trials daily and quickly learned many kinds of visually directed behaviors. By simultaneously training and testing multiple subjects programmatically, we were able to collect behavioral information at scale, allowing us to measure psychophysical tuning curves by changing parameters of the stimulus used to probe the behavior (Meier et al. 2011). We first measured the contrast tuning of orientation discrimination (*N* = 8 mice). The threshold contrast measured (~15%) was similar to thresholds measured earlier in mice (Prusky and Douglas 2004; Busse et al. 2011; Glickfeld, Histed, et al. 2013; Long et al. 2015), comparable with those of the hooded rat (Keller et al. 2000), but is an order of magnitude higher than contrast thresholds in monkeys and humans (De Valois et al. 1974). Nevertheless, mice improve performance with stimulus duration and thus are capable of evidence integration across time. This is similar to the behavioral responses in rats (Reinagel 2013) but potentially different from responses in monkeys and humans (Hanks et al. 2011).

One of the principal findings of this study is that mice can extract sufficient information on very short time scales (tens of milliseconds) to perform visually driven tasks. This is consistent with recent work in mice (Resulaj et al. 2018). Moreover, the useful integration times are an order of magnitude shorter than typical stimulus durations used to probe visual perception in mice (Andermann et al. 2010; Lee et al. 2012; Glickfeld, Histed, et al. 2013; Long et al. 2015). The total integration time—time beyond which subjects do not integrate visual information—was ~ 200 ms. This may not reflect an inability of mice to integrate information over longer time durations and might just reflect the strategy subjects use to maximize reward given the nonzero cost of accumulating evidence (Laughlin et al. 1998; Drugowitsch et al. 2012). Indeed, in other tasks, rats are known to integrate information over many seconds (Brunton et al. 2013). Nevertheless, subjects that integrate information quickly perform well in the orientation discrimination task.

The ability of mice to discriminate orientation requires V1, as their performance is compromised by optogenetic inhibition of V1 responses. This is consistent with the known response properties of V1 neurons (Hubel and Wiesel 1959; Niell and Stryker 2008) and with prior research in rodents (Glickfeld, Histed, et al. 2013; Resulaj et al. 2018) investigating the requirement of various visual pathways for perception. At these short time scales, we find that responses in V1 are sparse and unreliable. We find that the average neuron can fail to produce even a single spike to preferred orientations on 85% of the trials on average while still allowing maximal performance (85% correct) in subjects. The ability to discriminate orientation from responses of individual neurons based on a spike number code varied between 0.43 and 0.64 indicating that the best performing neurons do not come close to accurately predicting stimulus orientation. This stands in contrast with earlier work (Newsome et al. 1989; Vogels and Orban 1990; Britten et al. 1992) indicating that some V1 neurons can be as sensitive or more sensitive than the subject.

**Figure 5:**
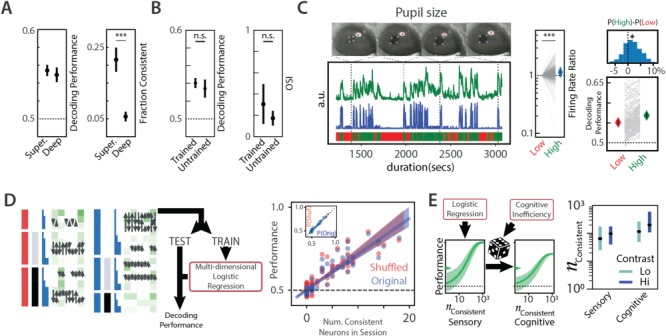
Contribution to decoding performance. (*A*) Contribution of depth. Decoding performance of consistent units (left panel) and fraction of units that were consistent (right panel) for deep and superficial units. (*B*) Contribution of training. Decoding performance (left panel) and orientation selectivity of units recorded in untrained versus trained animals. (*C*, left panel, bottom) *z*-score of pupil size (green) and running speed (blue) of a head-fixed mouse while viewing visual stimuli across an entire session. Blocks of low arousal (red lines) and high arousal (green lines) are marked below. Sample images of the pupil at different times during the session are shown (left panel, top). The corneal reflection is in red, while the extent of the pupil is shown in blue. Center panel: average firing rates of recorded neurons normalized to its firing rate during low-arousal trials. Diamonds indicate the average ratio across the population (***P <  0.001). Right panel: orientation decoding performance during low- and high-arousal epochs for consistent neurons (bottom) along with the histogram of the difference in performance between the 2 conditions (top). Diamonds are mean values, and error bars are 95% CI. Some error bars are too small to visualize. (*D*) Contribution of correlations. Left panel: schematic of shuffling strategy for the estimation of influence of correlations on performance. Stimuli were grouped according to stimulus parameters (orientation, contrast, duration). Spike number swaps (black arrows) occurred only between stimuli of identical parameters. Right panel: multidimensional logistic regression fits obtained as in Figure 4*B* for the original (blue) and shuffled (red) data as a function of the number of consistent neurons. Inset shows the performance on shuffled sessions plotted against performance on the original sessions. (*E*) Contribution of strategy. Left panel: Integrating evidence from a population of neurons is modulated by cognitive inefficiencies on random trials, leading to lapse in performance. Right panel: the number of neurons required to perform as well as the animal if lapses in performance could be attributed to sensory noise alone (sensory) or due to cognitive contributions alone (cognitive). Abbreviations: Hi, high; Lo, low; Num., number; Shuf, shuffled.

Why are response reliabilities and decoding power so different in the mouse? A variety of experimental factors may underlie this discrepancy. First, this difference could be due to the species used. Mice do not use vision as their primary sensory modality as opposed to primates and carnivores, in which similar stimuli may drive reliable and robust responses. However, high-contrast, 100-ms stimuli may not be the minimal discriminable stimuli in primates and carnivores, and responses to these minimal stimuli may be equivalently unreliable and sparse (Berens et al. 2012). Second, our results could be due to the location of our recordings. Our dataset contains neurons from all layers of the cortex. Separating the superficial responses (L2–L4) from the deep responses (L5–L6) indicated that, while the decoding performance of consistent neurons was comparable between the 2 layers (Fig. 5*A*, left panel), superficial layers were enriched in consistent neurons by ~ 4-fold (Fig. 5*A*, right panel). Third, it is possible that training in the orientation discrimination task may alter both the orientation tuning and the reliability of the neurons (Poort et al. 2015). To assess if training in the behavioral task improves the reliability of the neurons, we separated the responses of the cohort of animals (*N* = 6 mice, 16 sessions) that had experience with the orientation discrimination task. We found that training changed neither the decoding performance (Fig. 5*B*, left panel) nor the average orientation selectivity (Fig. 5*B*, right panel) in our data. Fourth, the low reliability in our recordings could be attributed to the uncontrolled and variable attentional state of the animal. It remained possible that, in an attentive state, similar to the state of the animal while performing behavioral trials, V1 responses would be more reliable. To assess if this was the case, we recorded from a small cohort (*n* = 4 animals, 38 session, *N* = 536 units) while simultaneously recording pupil diameter and running speed (Fig. 5*C*, left panel). Running has been shown to amplify V1 responses in mice (Niell and Stryker 2010), while pupil diameter is well known to modulate with attentional engagement (Reimer et al. 2014). We analyzed neural responses during intervals of high engagement (pupil diameter *z*-score > 0) and compared it with intervals of low engagement (pupil diameter *z*-score ≤ 0). This split the data to include 50% of the trials in the high-arousal subset (Fig. 5*C*, green lines). Intervals of high engagement led to both significantly higher firing rates (Fig. 5*C*, center panel) and a higher decoding performance in neurons (Fig. 5*C*, right panel) compared with intervals of low reliability. Nevertheless, the improvements in predictive power were small (~2% difference; Fig. 5*C*, right panel, top), and the responses were not reliable enough to predict the animal’s behavioral performance at a single neuron level.

Since the animal performs much better than would be predicted from single-unit responses, we sought to explore the impact of pooling information over populations of neurons on stimulus prediction. We estimate that pooling across ~ 75 consistent neurons would be required to perform as well as the subject. Prior estimates of the population requirements of neural coding have indicated that very few neurons may be required for certain kinds of sensory discriminations in mammals (Newsome et al. 1989; Britten et al. 1992; Stüttgen and Schwarz 2008; Huber et al. 2012), and indeed, responses to longer duration drifting gratings showed similar population requirements (see Supplementary Figure 3B,C). However, the minimal required population requirements measured using sparse optical activation were found to be comparable with those measured in this study (Huber et al. 2008). The mouse V1 contains ~ 1 million neurons (Leuba and Garey 1989; Schuz and Palm 1989; Collins et al. 2010) in each hemisphere. Thus, the orientation discrimination task requires only a small fraction of the overall population (<0.1%) for adequate performance.

In our analyses of how pooling leads to improved performance, we neglect the effect that correlations play in the cortical code. Neural responses within the cortex show a significant correlation with one another (Zohary et al. 1994; Kohn and Smith 2005). Under some scenarios, covariations in neuronal responses prevent pooling responses across multiple neurons from removing noise (Shadlen and Newsome 1998) and therefore might reduce the overall information available to the subject. Thus, it is possible that correlations across V1 neurons limit the total information the animal can extract from the population. Alternatively, however, correlations could make decoding stimuli easier if it is another channel to communicate information about the stimulus present in the animal’s environment. In this situation, including correlations might improve the code (Averbeck et al. 2006; Cohen and Kohn 2011; Froudarakis et al. 2014). We investigated the effects of correlation by artificially removing them from our recorded sessions, by shuffling neural responses across trials (Fig. 5*D*, left panel). Shuffling neither changed the slope of the “performance versus number of neurons” curve (Fig. 5*D*, right panel) nor the average performance by itself (Fig. 5*D*, right panel, inset), indicating a minimal role for correlations in affecting decoding performance for just-discriminable stimuli. However, even small correlations can affect the decoding of stimuli if information is spread across large populations of neurons (Zohary et al. 1994), and the precise structure of the correlations plays an important role in the decodability of population responses (Beck et al. 2011; Moreno-Bote et al. 2014). We have only tested the role that correlations play for population sizes between 1 and ~ 50 neurons. Based on the population requirement for reflecting behavioral performance (~75 reliable discriminators) and the measured prevalence of reliable discriminators (~1 in 10), we would then need to measure the influence of correlations on a simultaneously recorded population with a size of ~ 750 neurons. Future experiments should aim to record from such large populations and directly measure the effects of correlations on decodability. However, based on our existing dataset, correlations play only a marginal role in decodability.

Another assumption that could affect how integrating noisy evidence could limit the total information available for discriminating stimuli relates to the source of noise in the brain. Our model assumes that the noise in the sensory system limits the behavioral precision of the subject and the reason why performance plateaus for longer duration stimuli is due to the subject only integrating from a small subset of responses instead of using all the available information. However, cognitive inefficiencies could be an alternate source of noise in the system such that behavioral performance can be degraded even in the presence of perfect sensory information. We modeled these cognitive effects using a simple 2-step model: 1) Logistic regression of neural responses provided sensory evidence, which 2) on a random fraction of trials (equal to the lapse rate), was corrupted by the cognitive process (Fig. 5*E*, left panel). This led to reduced decoding performance for a given population size such that the subject would need to integrate from a larger pool of neurons to achieve the same decoding performance in the orientation discrimination task. Our data indicate that the population required to predict the orientation of the stimulus varied from ~ 75 for the sensory-noise–limited model to > 150 consistent units for the cognitive-noise–limited model. We expect that the actual number lies somewhere in the middle.

Finally, given the unreliability of individual neurons and the evidence that behavioral performance is dependent on pooling information from a population of neurons, it is worth asking how such pooling would be achieved in the cortex. Both L2/3 neurons and L5/6 neurons are known to receive information from L2/3 neurons and send projections to other parts of the neocortex. So, these are likely populations whose firing would reflect pooled population response. The fact that even the best performing L2/3 neurons are poor predictors of performance, even though previous data indicate that L2/3 neurons have a bias towards making connections with other L2/3 neurons with similar orientation selectivity (Gilbert and Wiesel 1989; Malach et al. 1993), suggests that L2/3 may not be the site of population pooling. L5/6 neurons can be eliminated based on similar reasoning. The increased prevalence of consistent units in the superficial layers may indicate that information relevant to this orientation discrimination task may be broadcasted to other areas of the cortex preferentially through L2/3 neurons—a hypothesis that can be tested with layer-specific manipulation of neural or synaptic activity. However, our data indicate that the site of population pooling likely resides outside V1. Careful dissection of the visual pathway through optogenetic inactivation of secondary visual areas along with recording of neural responses in these areas may be needed to definitively identify the integrator. Nevertheless, this integrator must be capable of producing reliable outputs from the unreliable outputs of V1. Our findings provide evidence that visually guided behavior at the limits of perception relies on effective integration of information across units with sparse and unreliable responses to stimuli.

## Funding

Gatsby Charitable Trust (grant awards GAT3138 and GAT3213); Biogen Inc, Cambridge, MA, USA; and NARSAD Young Investigator Grant (to A.C.M.).

## Notes

We are grateful to Pamela Reinagel for support and feedback relating to this manuscript as well as for foundational work on automating visually-directed tasks in rodents. We would like to thank Daniel Bishop, Madeleine Ciobanu, Yash Gandhi, Ashley Gutierrez, and Julia Ramirez for assistance with behavior experiments and Robert Recatto for help with analysis.

## Supplementary Material

CC_Supplemental_Resubmission_r2_bhz147
