# Supplementary material for: A Sparse Probabilistic Code Underlies the Limits of Behavioral Discrimination
Source: Cereb Cortex. 2019 Aug 12;30(3):1040–55. doi: 10.1093/cercor/bhz147 (PMC7132908; doi:10.1093/cercor/bhz147)
Supplement: CC_Supplemental_Resubmission_r2_bhz147 [file cc_supplemental_resubmission_r2_bhz147.pdf]

A Sparse Probabilistic Code Underlies the Limits of Behavioral Discrimination

Balaji Sriram[1,3], Alberto Cruz-Martin[2], Lillian Li[1], Anirvan Ghosh[1,3]

Division of Biology, University of California, San Diego, La Jolla, California, USA[1]

Department of Biology, Boston University, Boston, Massachusetts, USA[2]

Research and Early Development, Biogen, Cambridge, Massachusetts, USA[3]

Corresponding author:

Anirvan Ghosh
Research and Early Development, Biogen
225 Binney St
Cambridge, MA 02142
Email: anirvan.ghosh@biogen.com
Ph: 617 679 2000

Running Title: Population Coding at the Limits of Behavior

Supplementary Figures

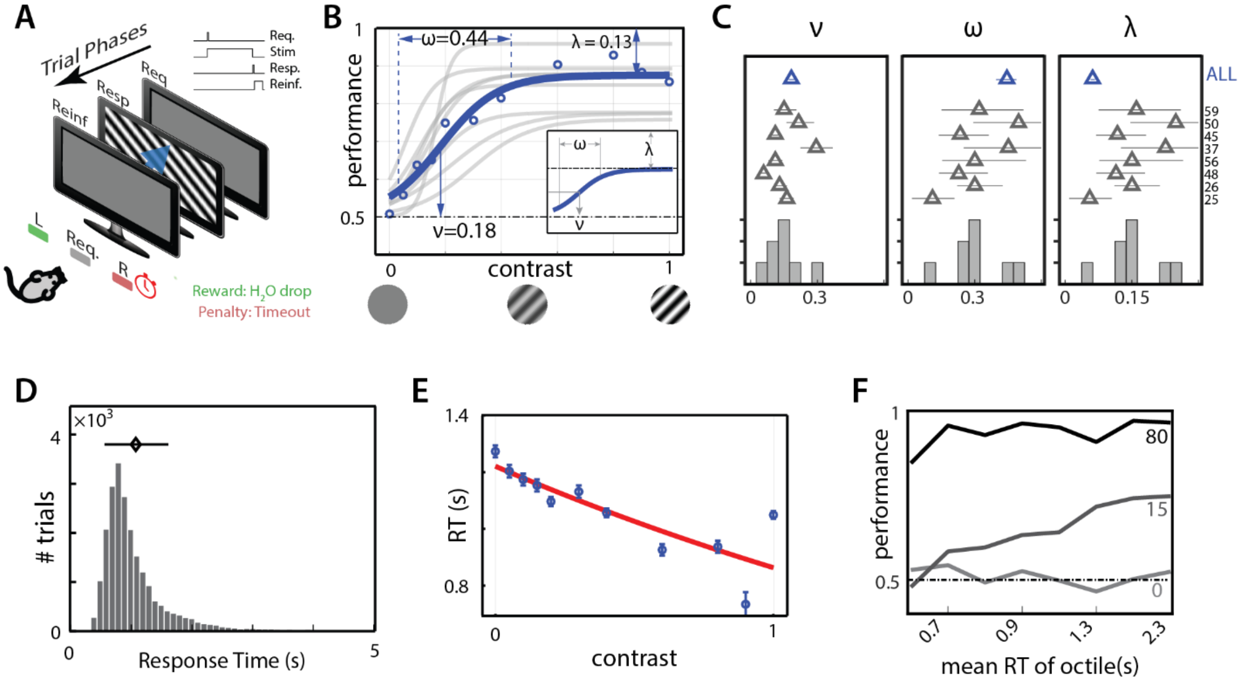

Supplementary Figure *1*- **Mice integrate visual informatio*n*.** **(A)** Schematic of trial structure for contrast dependence of orientation discrimination*.* **(B)** Performance improves with contrast. Sigmoid fits to performance of individual subjects (grey) and performance at specific contrasts along fits for the average mouse (blue) are shown. Also shown are the MLE of contrast at half-max performance (υ), tuning curve width (ω) and lapse rates (λ). **(C)** Fitted values of contrast at half-max performance (υ, left panel), contrast tuning width(ω, middle panel) and lapse rates (λ, right panel) for individual subjects (grey triangles with 95% CI), and for the average subject (blue triangle with 95% CI). Histogram at bottom shows the distribution of these fitted values. **(D)** Distribution of reaction times for the average mouse. Average reaction time (black diamond) with 1 SD shown. **(E)** Average reaction time as a function of contrast. Data show expected and 95 % CI of mean reaction times. Best exponential fit is shown in red. **(F)** Mean performance of the average subject for 80% (black), 15% (dark grey) and 0% (light grey) contrast as a function of the reaction time split into octiles.

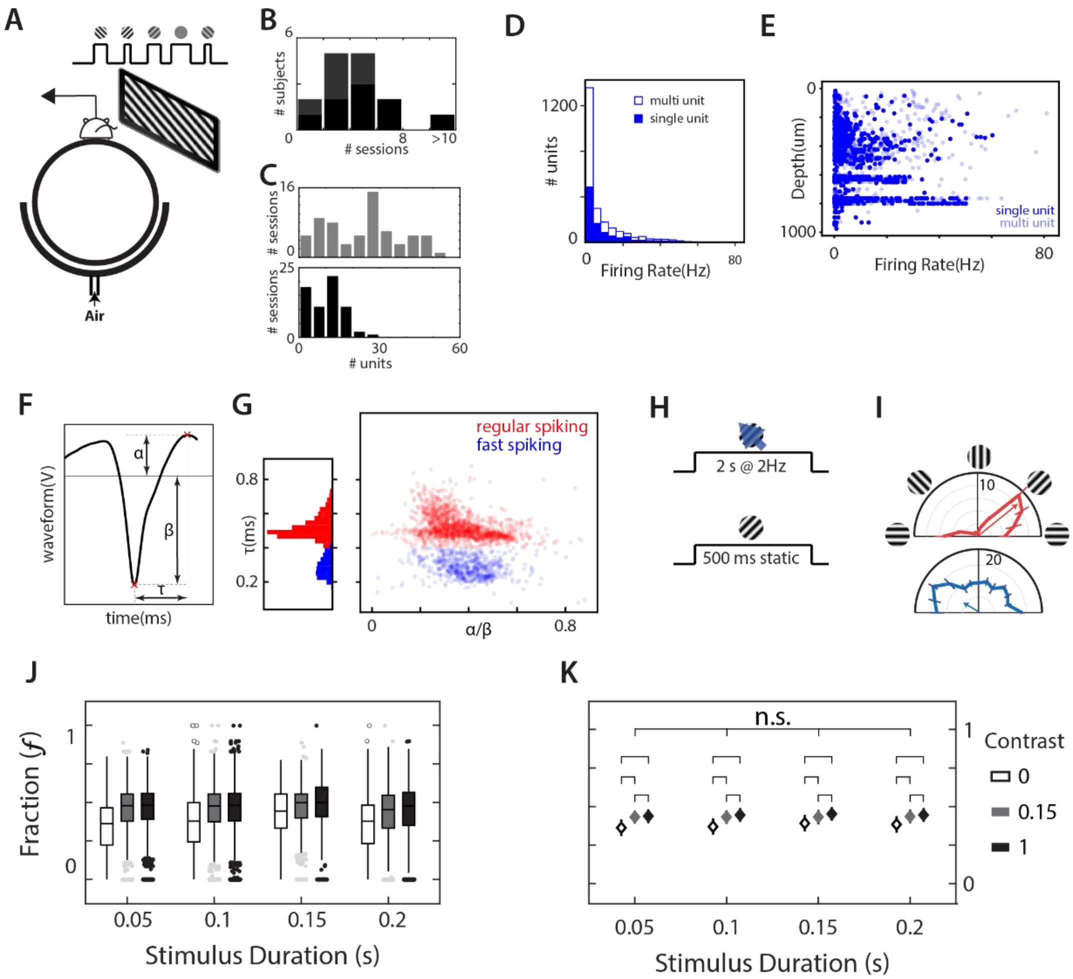

Supplementary Figure 2 - **Statistics of Recording in V1**. **(A)** Schematic of recording setup. (top panel) Subjects run on a Styrofoam ball suspended in air wile recording in the left primary visual cortex. We show visual stimulation to the right eye on an LCD monitor placed ~15 cm from the eye tangential to the eye. (bottom panel) Stimuli are gratings of different orientations, contrasts and durations separated by short periods (~1s) of gray screen. **(B)** Number of sessions collected from each subject (naïve: black; experienced: grey). **(C)** Distribution of total number of units (top panel) and well identified single units (bottom panel) collected in each session **(D)** Distribution of firing rates of single units (solid) and multi-unit activity (boxed) across all sessions. **(E)** Firing rates as a function of recorded depths. **(F)** Schematic of spike waveform. Red marks indicate the peak and trough of the spike. Measured features (α, β, τ) are marked **(G)** Distribution of the peak-to-trough duration (τ, left panel) and its relation with the peak-to-trough ratio (right panel). Individual neurons are separated into fast-spiking (blue) and regular-spiking (red) based on clustering analyses. Orientation tuning of individual units were measured using stimuli **(H)** that drifted for 2 seconds (top panel) or was static (bottom panel) allowing measurement of orientation tuning curves **(I)** of units having different orientation preferences . The distribution **(J)** and mean (K) of fraction of responsive neurons for all trials pooled across all sessions plotted as a function of contrast and duration of stimulus**.** Box plots indicate medians and quartiles. Outliers are plotted separately. Error bars are SEM.

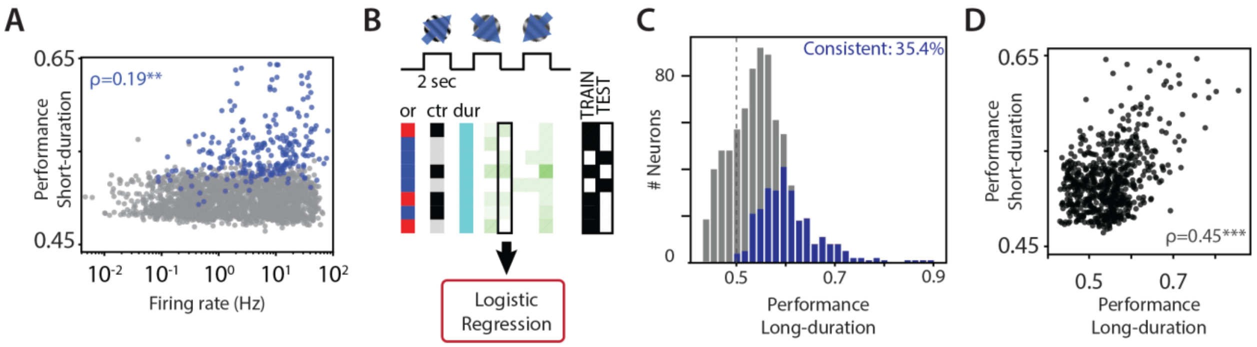

Supplementary Figure 3 – **Contribution of individual neurons to orientation decoding (A)** Decoding performance of units in the orientation discrimination task to minimally discriminable stimuli as a function of firing rates. Consistent units are plotted in blue. Performances of consistent units show a small but significant correlation with firing rate. **(B)** Schematic of logistic regression fit for long duration stimuli. The responses of the neuron were split into 70% training (TRAIN) and 30% testing (TEST) and spike counts were used as features for a logistic regression. Regression coefficients calculated from the TRAIN dataset was tested on the TEST dataset to obtain a decoding performance; experienced: grey). **(C)** Histogram of decoding performance of all (grey) and consistent (blue) units (calculated as in B, with spike counts over 2000 ms as features) in the recorded population. A performance of 0.5 indicates no information. **(D)** Performance of units to long duration stimuli plotted against performance to short duration stimuli. These show a small but significant correlation.

A

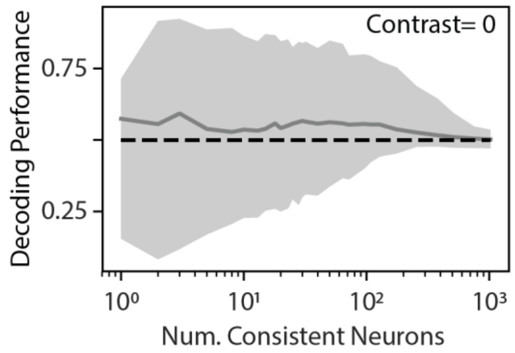

Supplementary Figure 4 – **Integrating evidence from a population of neurons (A)** Median (grey line) and 95% CIs (shaded area) of decoding performance of consistent units to stimuli of zero contrast in virtual sessions containing the responses from increasing population of consistent neurons. Chance level is shown as dashed line.

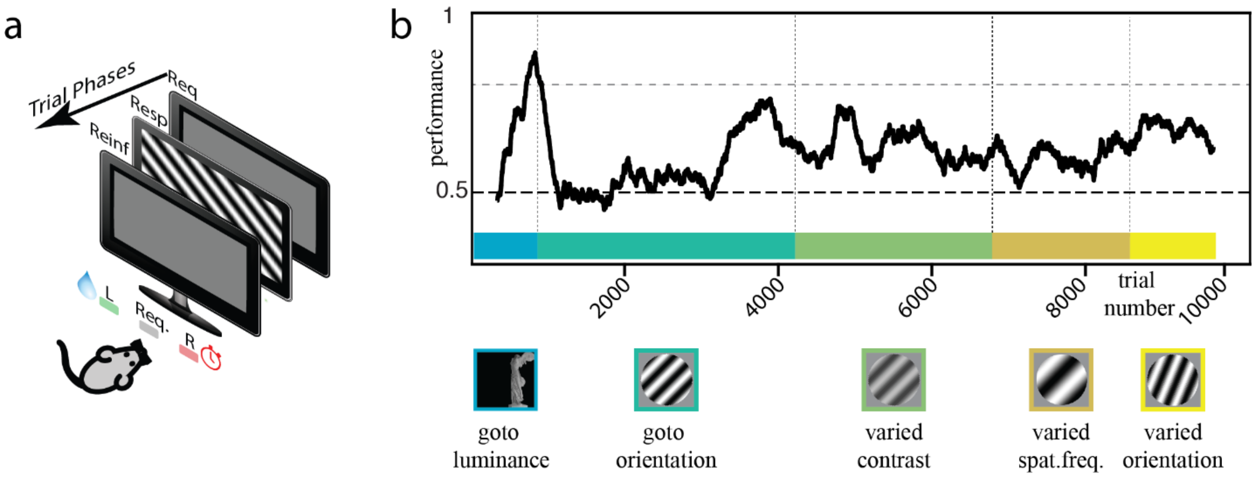

Supplementary Methods Figure 1: **Mice are trained in visual discrimination in an automated operant conditioning chamber**. **(A)** Schematic of trial structure performed by the subject. Subjects request trials during the Request phase (Req.) by licking the request port (center). Upon trial request, a visual stimulus is presented on the screen and the system waits for animal response (response phase; Resp.) The system then reinforces subject response in the reinforcement phase (Reinf.) by either providing water rewards (~10 ul) for correct responses or timeouts (5 – 20 s) for incorrect responses during which subjects cannot do any further trials. After the reinforcement phase, the system automatically returns to the request phase for the next trial. **(B)** Moving average of performance (black) of an example mouse through multiple steps (colored trial blocks) during the course of the experiment. Mice initially perform a go-towards-luminance task (blue). After reaching threshold performance, they are trained on a go-towards-orientation task (blue-green). After reaching threshold performance on this task, mice move into a series of modifications of the go-towards-orientation task where we varied various features of the stimulus(varied contrast, green; varied spatial frequency, orange; varied orientation, yellow) one at a time keeping all the others constant.

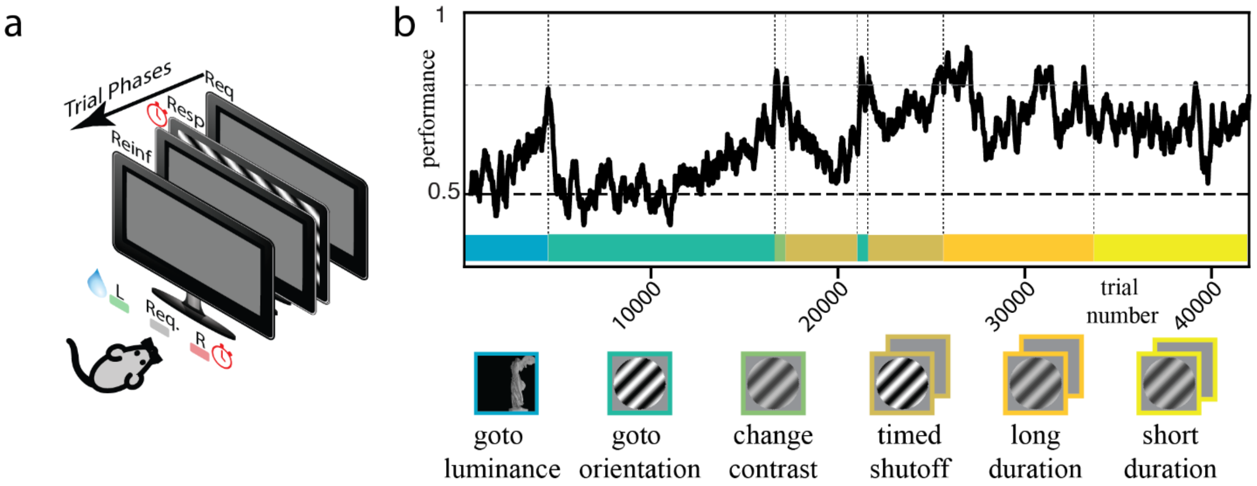

Supplementary Methods Figure 2**: Mice went through a series of steps to measure integration times**. **(A)** Schematic of trial structure performed by the subject. Subjects request trials during the Request phase (Req.) by licking the request port (center). Upon trial request, a visual stimulus is presented on the screen for a short duration before reverting to a grey screen and wait for subject response (response phase; Resp.) The system then reinforces subject response in the reinforcement phase (Reinf.) by either providing water rewards (~10 ul) for correct responses or timeouts (5 – 20 s) for incorrect responses during which subjects cannot do any further trials. After the reinforcement phase, the system automatically returns to the request phase for the next trial. **(B)** Moving average of performance (black) of an example mouse through multiple steps (colored epochs) during the course of the experiment. Mice initially perform a "go-towards-luminance" task (blue). After reaching threshold performance, they are trained on a "go-towards-orientation" task (blue-green). Subjects were then trained to perform OD on lower contrast trials "Change contrast" (green). In the timed shutoff step (brown) each trial request was followed by at most 1 second of high contrast stimulus following which the screen reverted back to gray screen. Subjects were forced to respond based on this limited information. After performing well on the times shutoff trials, subjects were shifted to trials where we varied the duration of the stimulus widely for durations between 120 ms – 2000 ms in the "long-duration" trials and between 16 ms – 250 ms in the "short-duration" trials. We manually moved the subject to an earlier task (at ~22000 trials) to improve motivation. The subject was shifted back to the timed-shutoff trials after ~1000 trials.

Supplementary Table 1: Training steps for basic characterization of orientation discrimination.

#	NAME	GOAL	STEP DETAILS	GRADUATION CRITERION	FIG. REF.
**1**	Free drinks	Learn to use reward ports	Reward ports occasionally squirt water	2 days	NA
**2**	Earned drinks	,,	Mice need to lick different ports to receive water.	3 days	NA
**3**	Luminance discrimination	Learn 2AFC trial structure	Large, high luminance stimulus appears on left or right side of monitor. Rewards only at side having high-luminance	80% performance over the previous 200 trials	Supp. Figure 1b (blue trial blocks)
**4**	Optimal Orientation discrimination (OD-optimal)†	Learn OD on simple high contrast gratings	Circular patch of drifting gratings (2 Hz, 0.08 cpd, 100% contrast) tilted left or right (45°) to vertical. Rewarded in the direction of tilt.	75% performance over the previous 200 trials	Supp Figure 1b (blue-green trial block)
**5**	Varied contrast†‡	Perform OD with varied contrast	Same as Step 4, except contrasts varied: $c \in [0,1]$	Manual graduation	Supp Figure 1b (green trial block)
**6**	Varied Spatial frequency†	Perform OD with varied spatial freqs.	Same as Step 4, except spat. freqs. varied: $sf \in \{0.03, 0.06, 0.13, 0.25, 0.5, 1\}$	Manual graduation	Supp Figure 1b (orange trial block’
**7**	Varied Orientation†‡	Perform OD with varied orientations	Same as step 4, except orientations varied: tilted $\theta \in [0, \pi/4]$ from vertical.	Manual graduation	Supp Figure 1b (yellow trial block)

†: All Orientation discrimination stimuli had randomized orientation presented on every trial. The direction of drift was also randomized

‡: These steps contained completely ambiguous trials (0 contrast in step 5 and 0 in step 7). The correct response port was decided based on the nominal orientation of the stimuli (tilted right with 0 contrast or 0° gets rewarded in the right port).

Supplementary Table 2: Training steps for measuring integration time.

#	NAME	GOAL	STEP DETAILS	GRADUATION CRITERION	FIG. REF.
**1**	Free drinks	Learn to use reward ports	Reward ports occasionally squirt water	2 days	NA
**2**	Earned drinks	,,	Mice need to lick different ports to receive water.	3 days	NA
**3**	Luminance discrimination	Learn 2AFC trial structure	Large, high luminance stimulus appears on left or right side of monitor. Rewards only at side having high-luminance	80% performance over the previous 200 trials	Supp. Figure 2b blue trial blocks)
**4**	Optimal Orientation discrimination (OD-optimal)†	Learn OD on simple high contrast gratings	Circular patch of flashed gratings (0.08 cpd, 100% contrast, random spatial phase) tilted left or right (45°) to vertical. Rewarded in the direction of tilt.	85% performance over the previous 200 trials	Supp Figure 2b (blue-green trial block)
**5**	Change contrast†	Perform OD at high or threshold contrast	Same as Step 4, except contrasts varied randomly $c \in \{0.15,1\}$	85% performance over the previous 200 trials	Supp Figure 2b (green trial block)
**6**	Timed shutoff†	Adjust to limited information	Same as Step 5, maximum duration of stimulus is 1s.	85% performance over the previous 200 trials	Supp Figure 2b (brown trial block’
**7**	High duration trials†	Perform OD with varied durations	Same as Step 6 but maximum duration varies between 128 and 2000 ms.	Manual graduation	Supp Figure 2b (orange trial block)
**8**	Low duration trials†	Perform OD with varied durations	Same as Step 6 but maximum duration varies between 16 and 500 ms.	Manual graduation	Supp Figure 2b (yellow trial block)

†: All Orientation discrimination stimuli had randomized orientation presented on every trial
